# The double agents in liquid biopsy: promoter and informant biomarkers of early metastases in breast cancer

**DOI:** 10.1186/s12943-022-01506-y

**Published:** 2022-04-04

**Authors:** Vinitha Richard, Matthew G. Davey, Heidi Annuk, Nicola Miller, Michael J. Kerin

**Affiliations:** Discipline of Surgery, School of Medicine, Lambe Institute for Translational Research, National University of Ireland, Galway, Ireland

**Keywords:** Liquid biopsy, Biomarkers, Breast cancer, Metastasis, microRNAs, Circulating tumor cells, Cell-free nucleic acids

## Abstract

Breast cancer continues to be a major global problem with significant mortality associated with advanced stage and metastases at clinical presentation. However, several findings suggest that metastasis is indeed an early occurrence. The standard diagnostic techniques such as invasive core needle biopsy, serological protein marker assays, and non-invasive radiological imaging do not provide information about the presence and molecular profile of small fractions of early metastatic tumor cells which are prematurely dispersed in the circulatory system. These circulating tumor cells (CTCs) diverge from the primary tumors as clusters with a defined secretome comprised of circulating cell-free nucleic acids and small microRNAs (miRNAs). These circulatory biomarkers provide a blueprint of the mutational profile of the tumor burden and tumor associated alterations in the molecular signaling pathways involved in oncogenesis. Amidst the multitude of circulatory biomarkers, miRNAs serve as relatively stable and precise biomarkers in the blood for the early detection of CTCs, and promote step-wise disease progression by executing paracrine signaling that transforms the microenvironment to guide the metastatic CTCs to anchor at a conducive new organ. Random sampling of easily accessible patient blood or its serum/plasma derivatives and other bodily fluids collectively known as liquid biopsy (LB), forms an efficient alternative to tissue biopsies. In this review, we discuss in detail the divergence of early metastases as CTCs and the involvement of miRNAs as detectable blood-based diagnostic biomarkers that warrant a timely screening of cancer, serial monitoring of therapeutic response, and the dynamic molecular adaptations induced by miRNAs on CTCs in guiding primary and second-line systemic therapy.

## Key points

- ❖ Tumor cells escape into the circulation at an early stage of transformation.
- ❖ Metastatic tumor cells may be single cells or a polyclonal cluster of leader cells that differ in mutations and gene expressions from the primary tumors.
- ❖ The secreted cell-free nucleic acids, small microRNAs, circulating tumor cells and proteins act as early diagnostic biomarkers of breast tumor.
- ❖ CTCs, CTC-specific microRNAs and circulatory miRs are collectively predictive of an early metastatic spread with prospective implications as therapeutic tools for targeting metastatic cells.

## Introduction

Cancer is the leading cause of death worldwide, with the incidence of female breast cancers (BCs) now surpassing lung cancer, with an estimated 2.3 million new cancer incidences and 685,000 deaths worldwide in 2020 [1]. Though this era is marked by advancements in precision diagnostics aiding an early detection of primary tumors (PTs), evidences of the presence of the undetectable systemic micrometastases that diversify even before surgical resection and later revert to recurrence, remains a persistent challenge [2]. Despite implementing best therapeutic strategies, nearly 20–30% of patients report clinical presentations of distant metastases arising from a single metastatic lesion which are capable of involving multiple organs [3, 4]. Early detection of metastatic breast cancer (MBC) entails screening for an upsurge in the levels of circulating protein tumor markers, carcinoembryonic antigen (CEA), cancer antigen (CA)-15–3, and CA-125 in liaison with simultaneous whole-body imaging [3]. The baseline expression value for these serum biomarkers varies across patients and the positive expression of few of these markers under the influence of anti-hypertensive medications and in inflammatory diseases affecting the liver, kidneys, and other organs have the capacity to impede theutility of these biomarkers in predicting MBC accurately [3]. Alternatively, the metastatic potential of node-positive and node-negative breast tumors is evaluated based on the protein levels of serine protease urokinase plasminogen activator (uPA) and the plasminogen activator inhibitor type-1 (PAI-1) in fresh or frozen tumor tissue of size not less than 0.125 cm^3^ which is impractical in unresected tumors of early breast cancer (EBC) [5, 6]. This underscores the need for a highly sensitive, non-invasive, and specific biomarker from an easily accessible source such as blood for the timely detection of metastatic spread and inception of therapy against the early metastatic clusters as well.

Tumor cells detach from the primary tumor bed and migrate to the circulatory system as singular units or clusters termed as circulating tumor cells (CTCs) [7]. These CTCs represent a minor fraction of early metastatic cells or a predominant set of late stage metastatic tumor cells that are latent with the potential of implantation at distinct organs even at an earlier time point of the disease [7, 8]. The concept of CTCs in blood was first described by Thomas Ashworth in 1896 [9] and nearly a century later Dr. Bernie Fisher (1965) proposed “the systemic or Fisher hypothesis of breast cancer” stating that “breast cancer is a systemic disease with malignant cells likely to be disseminated throughout the body before diagnosis” [10–12]. The current trend of cancer research addresses the “hypothesis of oligometastases” proposed by Hellman and Weichselbaum (1995) which state that “a limited number of metastases may represent a clinically significant disease state in which the full metastatic potential is not reached” [13]. The emerging molecular biology techniques have enabled the detection of oligometastatic CTCs in peripheral blood alongside the cellular by-products such as cell-free circulating nucleic acids (cfcNAs) - (ctDNA and ctRNA - miRNAs, microvesicles, exosomes) and circulating proteins collectively deemed as the “liquid biopsy” (LB) [7, 8, 14]. CTCs and the biomarkers involved have implications in detection of cancer, prediction and monitoring of therapeutic responses and stratification of patients to guide therapeutic cancer management [7, 8, 14–20].

Highly proliferative and aggressive breast tumor subtypes typically possess regions of enhanced apoptosis or necrosis subsequent to nutrient depletion, growth inhibition, and hypoxic conditions prevalent within the tissue core, which in turn releases nucleic acids (ctDNA and ctRNA) into the local tissues and circulation [17]. Despite these ctDNAs retaining their intrinsic mutation profile and the tumor-specific genetic and epigenetic aberrations of the breast tumor subtype, they may misleadingly represent the genomic status of dead cells and not that of the surviving fraction of highly metastatic tumor cells [18]. The diminishing concentrations of these components in bodily fluids is a major limitation to translational research efforts evaluating these biomarkers, which is technically challenging to overcome.

In the past two decades, there has been an increase in the research interest surrounding short (25 nucleotide (nt) long), single-stranded noncoding RNAs known as mi(cro)RNAs as abundantly expressed regulators of the transcription of protein coding genes [21]. MiRNAs are relatively stable and evade the enzymatic cleavage activity of the RNases which are abundant in the blood [22, 23]. The functional roles of miRNAs are versatile with a binary role in the maintenance of normal epithelial homeostasis as well as in the process of driving the oncogenic transformation. Moreover, they act as promising biomarkers which are highly informative in differentiating between normal and tumor subtypes, while remaining more stable than messenger RNA (mRNA) and ctDNA [24]. MiRNAs have great potential in the early diagnosis of multiple cancers, in accurate classification of poorly differentiated tumors, detection of tumor metastases, recurrence, prognosis and biological heterogeneity [24–34]. Interestingly, the secretion of miRNAs into the interstitial fluid (IF) and the tumor microenvironment (TME), also favours its utility as a blood-based biomarker for the early detection of metastatic cells [34, 35].

The objective of the current review is to address the molecular mechanisms that trigger an early undetected metastatic event and the genomic variations between early and late stage metastatic tumor cells. Secondly, we provide an updated view on the survival mechanisms adapted by these CTCs in circulation. Third, we elaborate on the additional biomarker components in blood that are disease-specific and explain how circulating and CTC-specific miRNAs may be considered as a sensitive and superior minimally-invasive liquid biopsy biomarker for accurate diagnosis of early metastatic CTCs. Following this we summarize on miRNA mediated targeted therapies against breast cancer metastases and the prognostic implications, the limitations encountered and conclude by suggesting promising future directions.

### The nuances of early metastases in breast cancer

The notion that fully transformed late disseminating tumor cells (DTCs) alone are able to initiate metastases is dismissed by studies that state even early transformed breast cancer cells (EBCs) with the crucial genetic and epigenetic mutations that reverse cell dormancy, are also capable of progressing to metastases [36, 37]. These EBCs need not originate from large tumors and may undergo metastases 5–7 years prior to the diagnosis of PTs [38]. The phenomenon of asymptomatic, clinically undetected minimal residual disease (MRD) or dormant DTCs attribute its origin to an early migration of small clusters (CTCs) from the original cancer or emerge as recurrence following an extended period of latency [39]. Though renamed for practical purpose, another variant to CTCs (also known as disseminated tumor cells (DTCs)) serve as reservoirs of small, dormant seeds that are capable of re-entering the vasculature and set-off a cycle of secondary spread throughout the body [40, 41].

Emigration of PT cells at an earlystage require disruption of the underlying basement membrane which is a major detectable sign of invasion [38]. Electron microscopic imaging reveals that few of the hyperplastic tumor cells of atypical ductal hyperplasia (ADH) lesions are capable of breaching the basement membrane by inducing localized secretion of proteolytic enzymes which is activated as a part of the gene regulatory program driven by the transcription factor Twist [38]. Of note, not all patients with detectable CTCs/DTCs in circulation develop metastases; previous studies have observed that CTCs/DTCs may remain in a state of dormancy without any clinical evidence of recurrence for up to 10–22 years until unknown mechanisms trigger the transition to overt metastases [42, 43]. The research community is still oblivious of the molecular mechanisms governing the dormancy of DTCs, the re-awakening cycles of MBCs and the identity of CTC subsets that are the true malignant seeds that can spread the disease.

#### Genomic variations between EBCs and metastatic clones

Meta-analysis of multiple tumors reveal that gene expression signatures of metastases differs considerably from their PTs and a uniform expression pattern is displayed by metastatic cells homing at the same secondary organ [34]. The location of secondary seeding plays a significant role in framing the genomic landscape and the malignant phenotype of metastatic cancer cells as the intrinsic molecular signature continue to evolve throughout its life history [34]. Almost 57% of cytokeratin-positive (CK+) single DTCs from the bone marrow of M0-stage breast cancer patients displayed significantly fewer and randomly generated chromosomal aberrations than PTs or cells from M1-stage patients that harboured different chromosomal imbalances typical of late metastatic cells [43]. Accrual of approximately 15,600 mutations in 65% of tumor cells have led to the emergence of dominant metastatic clones from the EBC primary tumor clones, imparting an unlimited proliferation potential and the ability to form metastatic tumor mass at distinct sites [7, 44]. Chromosomal imbalances are associated with hyper-proliferation in morphologically defined EBC lesions and occurs more frequently during the transition from ADH to carcinoma in situ (CIS), than in invasive cancers [45–47]. Furthermore, gene expression profiles of early primary breast cancers have differed from brain metastases (BM) with significant downregulation of immune-related gene signatures [48]. A parallel study affirmed that breast CTCs associated with BM also share the copy number alterations (CNA) of PTs, suggesting that BM competent cells have undergone clonal selection [49]. The overall indication is that CTCs from advanced stage breast cancers resemble the mutational profile of PTs more than the CTCs released at an early stage of breast cancer.

The Norwegian Women and Cancer (NOWAC) Post-Genome Cohort study compared the blood gene expression profile of healthy controls for up to 8 years before being diagnosed with early stage breast cancer and ascertained the supportive link between pathways regulating the immune system and metastatic spread [50]. The blood-borne CTCs of metastatic patients differed in expression of 55 mRNAs and 9 miRNAs from the CTCs of PTs and blood samples which is indicative of a molecular selection pressure exerted by minimally expressed miRNAs that are responsible for the emergence of receptor negative metastatic tumor cell fractions from estrogen receptor positive (ER+) PT cohort [51]. Nearly 80% of metastases from early-stage, low-density lesions are derived from early DTCs with the characteristic features of low proliferation, enhanced stemness, and a higher rate of migration supported by epithelial mesenchymal transition (EMT) [52], in comparison to advanced late-stage tumors that are more dense with proliferative cells and lower migratory potentials [53]. This shift from migration to proliferation is synchronised by a miRNA-mediated reversible downregulated expression of hormone receptors (HR) [53].

Genetic variations, mainly single nucleotide polymorphisms (SNPs), have been identified in miRNA and in the miRNA binding sites on target genes that may alter an individual’s susceptibility to breast cancer [54]. A SNP in the miRNA binding site is predicted to abolish miRNA–mediated silencing of the target mRNA leading to an upregulated translation of the corresponding encoded protein [54]. Amongst the five significant targets affected by the miRNA-associated SNPs, the master regulator of miRNA biogenesis DROSHA is the core nuclease that processes primary (pri-miRNAs) to precursor (pre-miRNAs) in the nucleus and a failure in suppressing Drosha leashes a massive synthesis of mature miRNAs that dynamically deregulate the normal tumor suppressor gene (tsg) signaling pathways and thereby increase the risk of breast cancer [54, 55]. Though the survival mechanisms adapted by tumor cells requires an intrinsic communication with the tumor microenvironment, miRNAs are crucial regulatory molecules with wide control over multiple genes and eventually proteins thus ensuring the existence of tumor cells in a dynamic host environment.

### Adaptations of circulating tumor cell clusters (CTCs) in blood

A metastatic tumor is a clonal representation of the PT if seeded by a single cancer cell and results in polyclonal metastases if seeded by a cluster of CTCs [56]. The conventional model of metastasis proposed that each metastasis is from a single tumor cell. However, salient discovery annulled the notion and showed that metastasis develops from a collection of multiple genetically distinct clones or polyclones [57]. The biological phenomena that triggered the discrete shedding of CTCs as single cells or as clusters from a primary cancerous lesion merely depends on the hypoxic status of tumor cells [58]. CTC clusters are highly enriched for tumor cells undergoing hypoxia, whereas single CTCs are largely normoxic and this intra-tumor hypoxia heightened the rate of CTC cluster shedding and formation of metastases [58]. CTC clusters also develop from oligoclonal tumor cell groupings by excessive production of cell junction component plakoglobin, which is known to arbitrate intercellular adhesion and increase the metastatic potential of tumor cells up to 50-fold [59]. Plakoglobin (or γ-catenin) interacts with several intracellular molecules including signaling proteins and transcription factors, which accounts for its involvement in cellular and oncological signaling [60]. A comparative gene expression analysis of CTCs, lung metastases and localized DTCs revealed that plakoglobin unified the cytokeratin 14 expressing (CK14+) oligoclonal CTC clusters, thereby imparting a low immunogenic profile that contributes to a successful dissemination [59]. In addition, the CK14+ tumor cells are found to be enriched in desmosome and hemidesmosome adhesion complex genes, co-expressed effector molecules of metastasis such as Epiregulin (Ereg), Tenascin C (Tnc), Jagged1 (Jag1) that are collectively involved in niche modelling and are deficient in major histocompatibility (MHC) class II genes, indicating a low immunogenic profile [57]. These CTCs also represent the aggressive CK14+ basal stem cell phenotype capable of colonizing distant organs efficiently [57]. CTC clusters are also found to be enriched in Ki67-positive proliferative cells that are epigenetically modified with activation of the stemness- and proliferation-related transcription factors Oct4, Sox2, Nanog and Sin3a, which also highlights the metastasis-seeding capabilities of CTC clusters than matched single CTCs [61]. Thus, CTC clusters are likely to be causative agents in inducing metastatic seeding of malignancies of the breast.

**Fig. 1:**
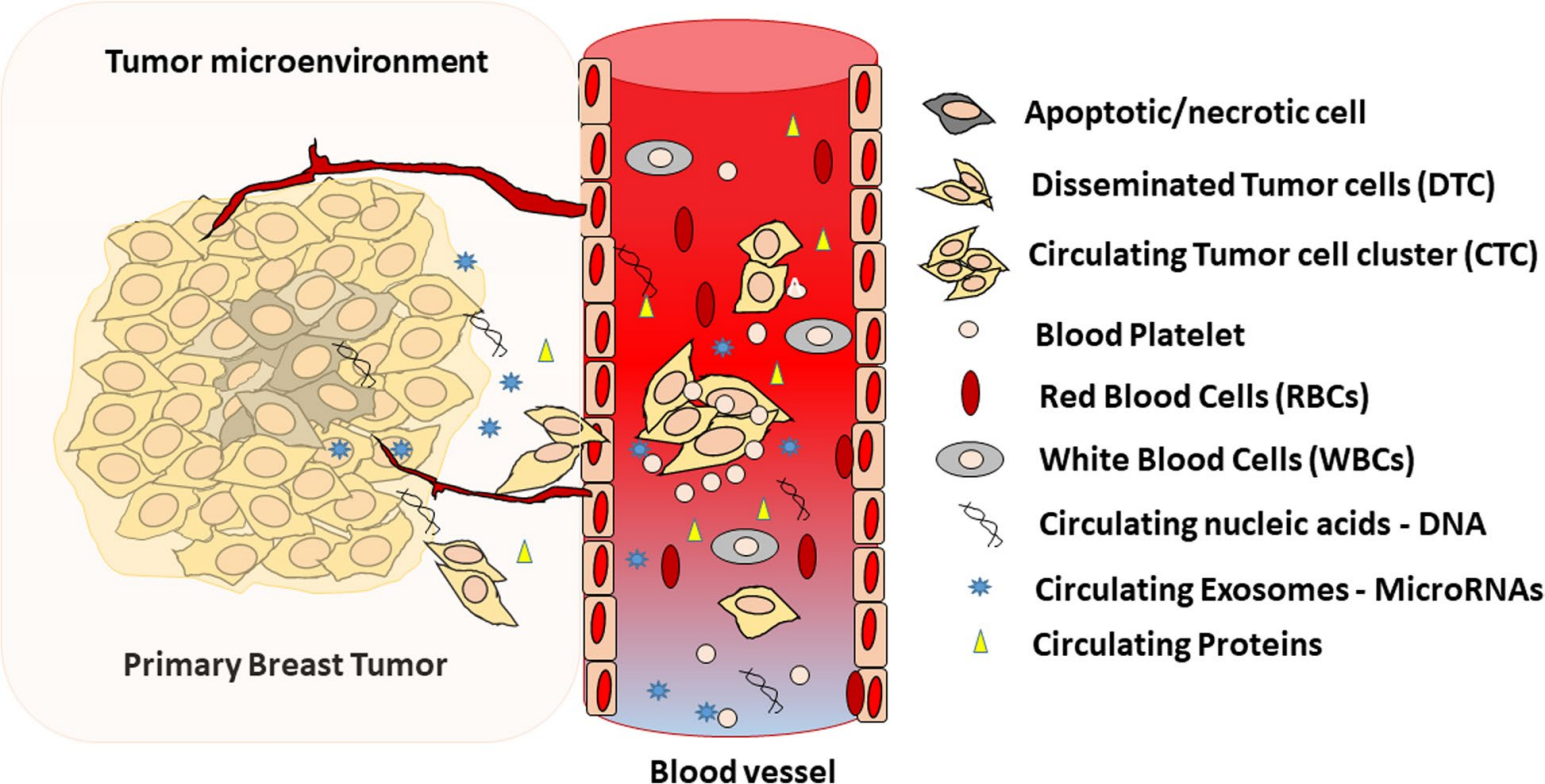
**Emergence of Circulating Tumor Cells in Early Stage Breast Cancer**. Tumor cells enter the blood stream as single CTC or cluster of CTCs endowed with attributes of immune evasion, stemness and proliferation potentials. These cells also secrete cell-free nucleic acids that may be utilized as blood-based biomarkers for detecting the presence of breast cancer

The onset of distant metastases originates with the translocation of CTCs into the blood or lymphatic system. This may occur through a passive mode with intermittent entry of cells into leaky blood vessels or by an active mode that strictly follows the central dogma of malignant transformation, starting with EMT, intravasation into adjacent blood vessels, migration through tissue layers or circulation, embolization, survival, extravasation and eventual re-seeding at a new site [57, 62]. Upon entering the circulation, the cluster signaling mechanism that drives the establishment of tumor outgrowth occurs through secretion of the growth factor “epigen”, which becomes concentrated in the ‘nanolumina’ intercellular compartments. An interruption in the production of epigen aborts the CTC cluster proliferation and promotes the transition of clusters to a state of collective migration [63]. Blood platelets also surround and cloak the CTCs forming a “microemboli” and in the process it imparts some protection against shear stress in the circulation [64]. This cloaking mechanism in turn serves to protect the CTCs from recognition and attack of immune cells in circulation, adding to the immune evasion hallmark of tumor cells in establishing a new niche (Fig. 1).

Studying CTC-clusters rather than individual CTCs is seemingly more informative in providing additional prognostic value with progression-free survival (PFS), especially in advanced-stage patients with inflammatory breast cancer (IBC), one among the most aggressive subtype of breast cancer which is associated with poor survival and outcomes [65]. Interestingly, there is a surge in the frequency of CTC-clusters during the course of neo-adjuvant chemotherapy (NAC), which profoundly declines post-surgery [66]. Despite surgical intervention, there remains a higher proportion of clusters in patients who have non-responding or resistant disease [66]. Longitudinal evaluation of changes in the cell count of single CTCs and CTC-clusters in patients with MBC tend to improve the prognostic value over time and indicate a successful monitoring during the first-line of systemic therapy [65, 67]. The incorporation of CTC-specific miRNA profiling as a combined prediction model also amplifies the prognostic accuracy of MBC patients and have utility in predicting pathological complete response (pCR) and in detecting responders to NAC in clinical practice.

The limiting factor in accurate detection of CTCs from whole blood is the inability of epithelial marker-dependent cell isolation methods to recognize transformed cells expressing mesenchymal markers. The remedy is to focus on cell morphology and utilize an epithelial marker-independent, unbiased enrichment or filtration technique that improves detection of CTCs and several such tests prove that CTC-clusters are more frequent in EBC than in MBC reinforcing the concept that metastasis is definitely an early event in breast tumor progression [67, 68]. The best alternative would be to resort to blood-based biomarkers that can specifically detect the presence of rare quantities of CTCs, CTC clusters and DTCs in any homeostatic conditions and at any time point of sampling.

### Multiple biomarker components of liquid biopsy in breast cancer

**Fig. 2:**
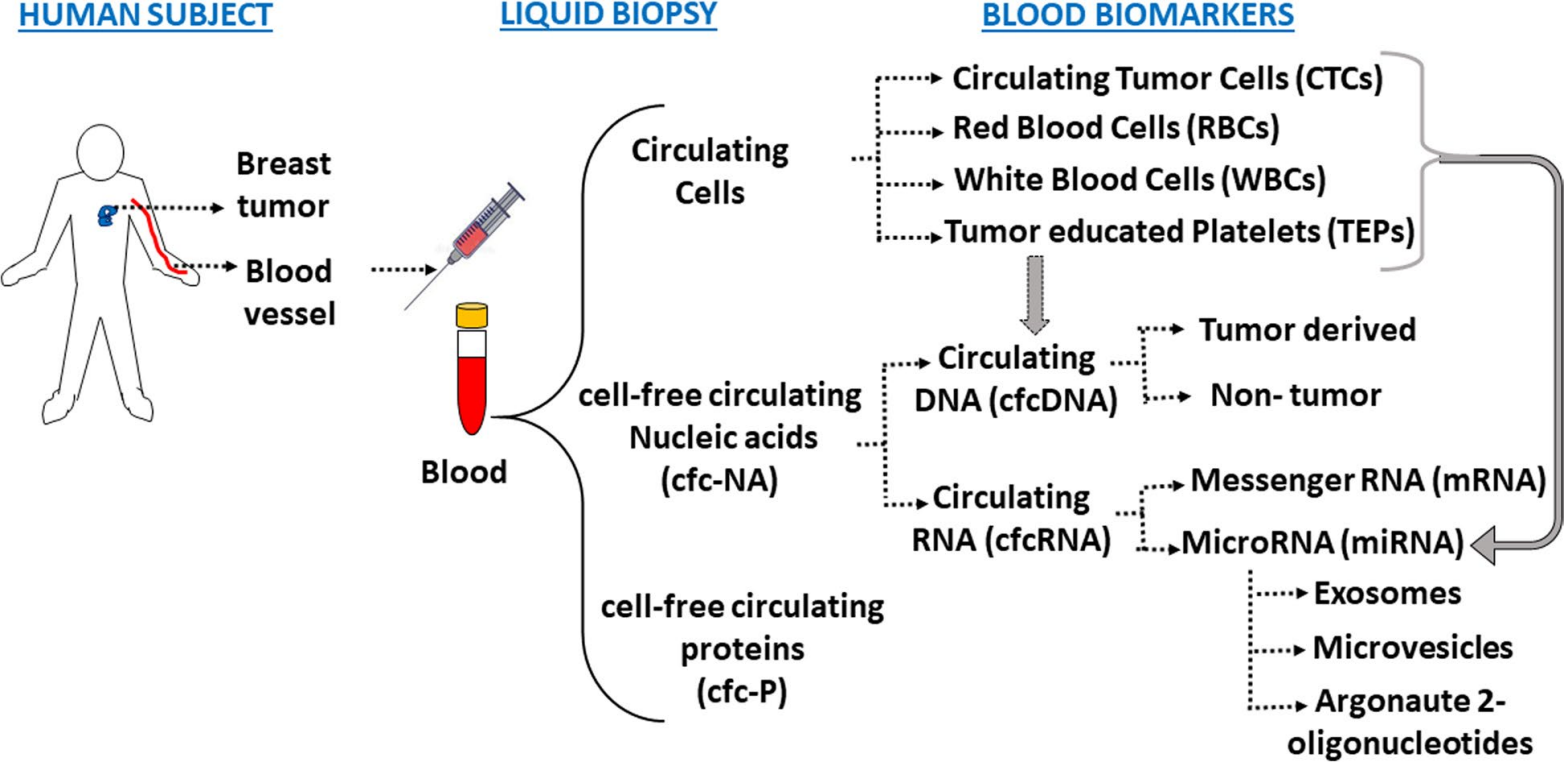
**Components of Blood Biopsy for the Early Detection of Breast Cancer**. Molecular components of liquid biopsy with immense implications in precision medicine

For decades, translational and clinical research efforts have been focused on the identification of molecular biomarkers capable of specifically distinguishing cancerous conditions from the healthy counterparts. Though tumor tissue biopsies provide a single preview of inherent tumor heterogeneity, it still falls short in accurate prediction of response to treatment. Liquid biopsies comprising of whole blood and other alternative body fluids such as urine, saliva, nipple aspirate fluid (NAF) and pleural effusions have previously overcome the limitations of painful invasive tissue resections [69]. Moreover, these offer a multi-modal reflection of the levels of tumor spread in the body, the associated cellular mutations, prediction of the emergence of drug resistance and an overall prognosis [69]. The predominant components of liquid biopsy are the factors released into blood stream by tumors such as CTCs, ctDNA, cfc-RNA (miRNAs and mRNAs), tumor-educated platelets (TEPs) and exosomes which show significant promise in pan-cancer diagnostics and in early-stage cancer detection [7, 8, 14, 70, 71] (Fig. 2).

#### CTCs and ctDNA in breast cancer

Detecting the presence of CTCs and ctDNA have become the standard therapy monitoring tools favouring sequential prognostic evaluation. Also the CTCs in blood delineate the temporal heterogeneity marked by gain or loss of HR expression which arises during the progression of localized tumors to metastases [72]. The presence of 1–10 CTCs per 7.5 ml of a blood sample is associated with decreased disease free survival (DFS), overall survival (OS) and in post chemotherapy patients, it is prognostic for poor relapse free survival (RFS) [73–76]. The maximum observed count of circulating cancer cells (CCC) per patient blood sample range from 2 for stage 1, 37 for stage 2, 19 for stage 3, and 1283 for stage 4 breast cancer patients clearly indicating that patients are more likely to have a metastatic disease if tested positive for CCC anytime during the sampling period [74].

HR status, tumor grade and the treatment modalities administered have previously illustrated no correlation to the presence of CTCs in stage 4 MBC patients [74]. Nearly 5% of HR–positive BC patients with a positive CTC assay displayed a 13-fold higher risk of late clinical recurrence (range: 0.1–2.8 years) when compared to negative CTC results. Despite similar CTC positivity, only 1 of 193 HR–negative patients (0.5%) displayed a late clinical recurrence at the time of the analysis [75]. CTCs and ctDNA are validated as independent markers of prognoses in MBC patients [76]. The question regarding how informative the presence of CTCs is remains since the spread of tumor cells is a phenomena known to be highly prevalent in advanced stages of the disease? Circulating cell-free DNA (ccfDNA) refers to any free DNA circulating in the bloodstream, regardless of origin and is higher in breast cancer patients compared to healthy subjects [77]. The short fragments (< 166 bp) of ccfDNA represent the tumor-originated DNA (ctDNA) [78] and the sequencing of ctDNA provide an updated mutational profile of the PTs, thereby enhancing individualized treatment options and longitudinal screening that aid in the decision-making to initiate adjuvant therapy to prevent recurrence [74].

A comparative screening of BC alterations in genomic DNA of tumor tissues and circulating free total nucleic acids (cfTNAs) from the plasma LB samples validated against the DNA from peripheral blood mononuclear cells (PBMCs), identified 7 disease-specific mutational variants in addition to *HER2* amplification in tissues and plasma [79]. Post treatment with targeted therapy using Trastuzumab Emtansine (T-DM1), most HER2+ clones (with *HER2* amplifications and *PIK3CA* mutations) disappeared allowing new passenger mutations (actionable *ESR1/PIK3CA* mutations and *MYC/FGFR1* amplifications) to appear in blood (during sequential LB) suggesting a systematic, molecular subtype switch to a new resistant (HER2-) clonal variant [79]. Moreover, there remains a significant risk of disease recurrence associated with the detection of CTCs in circulation, particularly in the setting of phospho-inositide3-kinase (*PIK3CA*)-positive disease [80–82]. Bulk sequencing and array of tissue-only analysis would miss this extensive remodelling at the molecular level that affects the dynamic oncogenic dependencies, but is now traceable through liquid biopsy. Thus, LB enables accurate identification of patients that respond to T-DM1 within weeks of therapy and potentially select patients who could be advised to resort to innovative pulse dosing/de-escalation schedules, with minimal treatment-associated toxicity [79].

An interesting finding is that the inception of spontaneous variations in specific cancer-associated genomic regions (CAGRs) are shown to harbour more than half of the reported human miRNAs [23, 83]. Genomic data analysis also proves that an amplification of DNA copy number aligns with an overexpression of miRNAs and genomic deletions correspond to the downregulation of miRNAs respectively [84]. This gives credit to the presence of an early set of secreted miRNAs as LB biomarkers as a sequel to gene mutations.

#### Circulating RNAs, exosomal RNAs and miRNAs in breast cancer

The PT cells and CTCs secrete cf-RNA/or ctRNA which includes mRNA and miRNAs that are encapsulated in exosomes which transform the tumor stroma and regulate multiple genes, thus facilitating the establishment of metastases [31]. Analysis of cf-mRNA in early-stage cancer patients provide valuable information regarding somatic mutations at the DNA level, gene expression profile of the tumor and also the epigenetic changes and other alterations in cellular pathways [73, 85]. MiRNAs are protected from ubiquitous ribonuclease (RNase) enzyme-mediated degradation by embedding in exosomes that are small membrane-bound extracellular vesicles (EVs) (30–200 nm), or in larger microvesicles (MVs) (250–400 nm) shed from the plasma membrane and also co-exist as free-form ctRNAs associated with a ribonucleoprotein complex or an argonaute-2 protein [22, 86–89]. Breast tumor secretory cells release microRNAs in microvesicles to regulate the gene expression of multiple neighbouring cells whereas miRNAs released by other cell types are as non-exosomal free oligonucleotides [90].

Although other non-coding RNAs (such as long-non-coding RNAs (lncRNAs)) act in unison with miRNAs as co-regulators of multiple protein-coding genes, lncRNAs mainly function as the molecular sponges of miRNAs by regulating the miRNA:target mRNA axis [91, 92]. Long non-coding RNA LINC00511 and onco-miR-301a-3p are found to be significantly overexpressed in early stage BC patients’ liquid biopsy samples and in patients with larger tumors (> 2 cm) as compared to later stages [92]. LINC00511 is also directly correlated with aggressiveness marked by Lymph node metastasis (LNM) and advanced tumor grade, whereas its downstream miRNA, miR-185-3p displayed a fourfold downregulated expression highlighting an independent mechanism of action suggesting that lncRNAs:miRNAs may also be utilized as tumor biomarkers in blood [93].

Extracellular RNAs (exRNAs) in circulation that are encapsulated within vesicles (EV’s) share the dimensions and density with the lipid components in circulation [86] which obscures the accurate identification and necessitates ultracentrifugation and chromatography techniques for purification of disease-specific EVs [94]. Combination of circulating EVs and CTCs are predictive of OS in MBC and other solid tumors [95]. The most important observation is that minute transitions in gene expression is reflected instantly at the transcriptional level. This wealth of information packaged into these exosomes maybe functional in eliciting a phenotypic response in recipient cells as in miRNAs and mRNAs [96]. Interestingly, miRNAs are found to be the most abundant exosomal RNA species, with nearly 593 detectable miRNAs that may have an important role in regulating biological functions, such as RNA splicing, protein phosphorylation, and angiogenesis [97]. Only a few highly abundant subset of cellular miRNAs are retained within the cell or are selectively released into the circulation and can be traced to the cell of origin [98]. These selectively released miRNAs (for example, miR-451 in breast cancer) are also found to be enriched in mammary fluids, blood and milk indicating that they are ideal diagnostic biomarkers for breast cancer and yet should be studied independent of the abundant cellular miRNAs [98]. A panel of circulating miRNAs (miR-141, miR-200a, miR-200b, miR-200c, miR-203, miR-210, miR-375, and miR-801) also predicted the CTC status of patients with MBC and previously differentiated the MBC cases from healthy controls [99]. Circulating miR-200b was identified as the best marker in distinguishing CTC-positive from CTC-negative patients [99]. The functional attributes of these selectively released miRs mediate the paracrine signaling between the tumor and stroma in promoting field cancerization [100].

### MicroRNAs as diagnostic and prognostic biomarkers in liquid biopsy

Secreted miRNAs in body fluids such as serum, plasma, and whole blood are promising candidates to function as cancer biomarkers for BC screening [101, 102]. Normal and tumor cells often contain intracellular pri-miRNAs that are more than 1000 nt in length, cleaved to pre-miRNA which is transported to the cytoplasm and pre-processed to functional (22 nt long) mature miRNAs [21, 96]. Only a few mature miRs have shown promise as biomarkers for the detection of malignancy in the plasma of BC patients and a recent study strongly highlighted the presence of plasma pri-miR526b and pri-miR655 as biomarkers distinguishing the plasma of stage I from benign conditions [102, 103]. Interestingly, molecular changes such as maturation and subsequent differentiation of PBMCs transpire even from the early-stages of neoplastic lesion formation, indicating that the miRNA repertoire in circulation modifies the TME thus favouring an early onset of metastasis [103–105]. A four miRNA signature panel (miR-145, miR-139-5p, miR-130a, and miR-425-5p) previously displayed the highest diagnostic accuracy for detecting early-stage BC patients [106]. Also the involvement of miRs in directing the progression of CTCs through every stage of metastatic events opens up new possibilities for tracking the malignant transformation at an earlier stage based on the expression of metastases promoting pre-miRs [31].

#### MiRNAs as prognostic biomarkers in post-operative BC patients

MiRNAs are currently established as minimally invasive whole blood diagnostic tools for early stage detection of PTs and metastases with immense practicality even in post-operative BC patients [107]. Analysis of gene copy number aberrations (CNAs) in a single circulating tumor microemboli (CTMs) and the relative PT tissue sample in early-stage breast cancer patients have supported the notion that CTMs are disseminated from genetically diverse and advanced regions of PTs [107]. Moreover, this suggests these cells are primed in advance with genes responsible for metastatic development [107]. A five-miRNA signature (miR-149, miR-10a, miR-20b, miR-30a-3p and miR-342-5p), previously used to substratify breast cancer patients who succumbed to early recurrence into a ‘high-risk’ group (with shorter RFS) and the subsequent miRNA-mRNA target prediction revealed an overall upregulation of proliferation and angiogenesis related gene expression [108]. Similarly, comparative screening of the expression profiles of miR-19a, miR-21, miR-22 and miR-127 performed across post-operative, non-metastatic EBC patients and healthy donors reflected the significant diagnostic values of these miRNAs in identification of EBC prior to or after treatment with adjuvant chemotherapy and at 2 years of follow up [101]. Although miR-21 is a well-described oncogenic miRNA found highly expressed in the circulation of breast cancer patients, it has been reported to be significantly downregulated in T0 patient blood samples, suggestive of an early deregulation of inflammatory and immune processes [101, 109]. Similar inferences have been reported regarding the circulation of miR-331 and miR-195 whose expression in plasma effectively differentiated the localized Luminal A tumor from metastases [110].

#### MiRNAs as mediators of molecular subtype transitions

Even clusters of miRNAs coded by the same chromosome (Ch19 miRs) (− miR-520 g, −520f, −520c, −520d, etc.) have been reported to be differentially expressed in breast tumor cells, serving as molecular classifiers of tumor tissue that is independently validated by artificial intelligence (AI) based network modelling and experimental research [27, 111, 112]. An overexpression of miR-520c and miR-373 appear to target the metastasis-related *CD44* gene, thus augmenting the invasion and metastasis of breast tumor cells [113]. The C19-miRs are also identified to target *REST*, *CEBPB* and *TFF3* genes which tend to increase the expression of a basal epithelial stem cell marker CK14, which is also a marker of polyclonal CTC clusters [59]. The expression of CK14 in tandem with the transient loss of *ERBB2* gene expression directs the molecular subtype switch of basal-HER2+ to the highly aggressive triple negative molecular subtype [114]. Notably, distinct yet interconverting phenotypes concur within CTCs in the same tumor subtype offering acquired drug resistance and evolution of MBCs [115]. CTCs in advanced ER+/HER2- primary tumors reflect a dynamic transition between HER2+ and HER2− subpopulations wherein HER2+ CTCs display a higher proliferation potential and HER2- CTCs exhibit a higher resistance to chemotherapy predicting that the TME (oxidative stress and cytotoxic chemo) define this spontaneous transition [115, 116].

#### Cell-free miRNAs and the targeted signaling pathways

A comparative study conducting miRNA profiling of liquid biopsies in healthy age-matched individuals previously revealed the differential role of cell-free miRNAs as well as the unique miRNAs packaged into exosomes targeting the neural and axonal development, TGF-beta signalling, and pathways in cancer [104]. Moreover, the intracellular miRNAs isolated from blood cells (RBCs, WBCs, platelets) are known to be profoundly enriched in miRNAs targeting self-renewal, focal adhesion, glioma, phosphatidylinositol and melanogenesis signalling pathways [104]. Plasma miR-1246 was the first microRNA isolated from EVs to be used to aid BC diagnosis [117]. Likewise, a three miRNA expression panel (miR-142-5p, miR-320a and miR-4433b-5p) measured in serum also accurately detected BC molecular subtypes [118], while another set of 4 differentially expressed ct-miRNAs in plasma (miR-20b-5p, miR-92a-2-5p, miR-106a-3p and miR-106a-5p) and in serum (miR-19b-3p, miR-20b-5p, miR-92a-3p and miR-106a-5p) successfully discriminated BC patients from control [119, 120]. Another study on LB reported an upregulated expression of miR-99a, miR-130a, miR-484 and miR-1260a in the plasma of BC patients and target predictions identified these miRNAs to be involved in regulating the Hippo and Transforming Growth Factor-beta (TGF-beta) signaling pathways [121]. A combination of only three miRNAs (miR-148b, miR-409-3p and miR-801) are found to be significantly upregulated in the plasma of BC patients, differentiating between BC cases and healthy controls [122].

Extracellular miRNAs should be considered as independent diagnostic markers of disease in comparison to cellular miRNAs as observed in the selective release of miR-1246 and miR-451 by BC cells and the concentration of these in body fluids during the transition from normal to abnormal cells [98]. The higher expression of hsa-miR-652-5p in pre-treatment plasma of BC patients also outlines it as a possible blood-based biomarker for early detection of breast cancer [123]. Another prominent systemic biomarker to discriminate EBC cases from controls is miR-195 [124]. A second study reported a downregulated expression of miR-195 in serum of early-stage BC patients with a much higher sensitivity of detection than conventional tumor markers CA153 and CEA [125]. Inconsistencies in expression levels, elusive targets and the mode of action are some of the hurdles associated with several of these systemic biomarkers.

Undetected metastases to secondary organs is a major cause for mortality and unfortunately 30–55% of those diagnosed with triple negative breast cancer (TNBC) and HER2+ breast carcinoma succumb to brain metastases (BM) [31]. Survival in such instances is dismal (median survival: 4–14 months) [31, 126, 127]. Disseminated tumor cells from PTs alone are not capable of executing this multi-step process of BM, particularly due to the presence of the blood–brain barrier (BBB). In circulation, the CTCs secrete miRNAs via exosomes which suppresses the immune system, transform the brain stroma, and breach the BBB for BM [31]. BM prone BC cells release EVs carrying miRNA-181c that targets the actin dynamics and eventually lead to the destruction of the intact BBB [128]. A proportion of BC patients may develop distant metastases to bone leading to formation of osteolytic lesions [129]. The bone-tropic ER+ breast cancer cells tend to secrete exosomal miR-19a and Integrin-Binding Sialoprotein (IBSP), thus enhancing the expression levels of these markers in circulation of ER+ BC patients [130]. IBSP attracts osteoclast cells to the bone and exosomal miR-19a induce osteoclastogenesis by remodelling the TME in a paracrine manner favouring BC cell colonization in the bone without directly affecting the cancer cells [130]. All these findings suggest that CTCs alone are incapable of establishing metastases to different sites, but require the assistance of circulating miRNAs to regulate the multiple cellular and molecular events promoting the advancement of BC.

#### MiRNAs as predictors of therapeutic resistance

In the translational research arm of the prospective, multicentre neoadjuvant NeoALTTO trial, increased expression of plasma ct-miR-148a-3p, ct-miR-374a-5p and ct-miR-140-5p correlated with a 54% increase in pCR in patients being treated with neoadjuvant trastuzumab-based therapy for HER2+ disease within two weeks of initial treatment [131]. Non-responders to NAC in TNBC are identified through varying expression levels of blood-derived exosomal miRNA biomarkers, marked by a reduced expression of miR-185, miR-4283, miR-5008, and miR-3613, as well as upregulated levels of miR-1302, miR-4715 and miR-3144 [132]. The target genes of these miRNAs belong to pathways typically related to immune response activation and suppression, highlighting a defective immune system may be responsible for resistance to NAC in TNBC patients [132]. Interestingly, the measurement of a five miRNA expression panel (miR-1246, miR-1307-3p, miR-4634, miR-6861-5p and miR-6875-5p) in serum, accurately detected EBC with 97% sensitivity from the non-BC patients [133]. The SUCCESS clinical trial previously investigated the presence of plasma miRNAs in BC patients pre- and post-chemotherapy against healthy controls [134]. This trial observed significantly increased plasma levels of miR-16, miR-27a and miR-132 in BC patients prior to chemotherapy versus controls, while levels of these biomarkers later reduced to match the levels observed in healthy women post chemotherapy [134]. Therefore, it is reasonable to suggest that these miRNAs have oncogenic properties and may be useful in gauging treatment response to NAC. Additionally, expression levels of miR-16, miR-107, miR-130a and miR-146a have been previously reported to substratify patients with lymph node positive and lymph node negative disease [134]. The levels of miR-130a and miR-146a also differed – based on HER2 status suggesting a potential role of these miRNAs in the HER2 signaling pathway in these biologically distinct cancers [134].

Analysing the plasma levels of miR-923 and CA-15-3 protein in combination with routine clinicopathological data has clearly predicted disease recurrence and prognosis in preoperative patients, irrespective of the treatment regimen [135]. Screening for an upregulated expression level of serum exosomal miRNAs (miRNA-21, miRNA-222 and miRNA-155) identified patients who failed to respond to NAC and subsequently developed distant disease [136]. These studies also emphasize the collective use of exosomal miRNAs, cell-free proteins, and CTCs as both diagnostic, prognostic, and predictive biomarkers in BC (Table 1). The technical obstacles associated with the comprehensive tracking of relatively minor changes in expression levels of molecular biomarkers that originate with the release of a single or multiple CTCs and monoclonal or polyclonal CTC clusters in the blood stream remains to be determined.

**Table 1:** Cumulative list of circulating biomarkers in liquid biopsies of early and metastatic stage breast cancer patients

No.	Clinical Study	Endpoints	Source	Samples	Method of detection	Biomarkers	Ref
1.	CTC-specific miRNAs	Enhance the prognostic accuracy of CTCs in MBC	LB – CTCs (peripheral blood)	MBC with CTC ≥5/7.5 mL (*n* = 16); patients with CTC = 0/7.5 mL (*n* = 16) and healthy donors (*n* = 8)	CellSearch system for CTC detection	⬆ CTC-specific miR-106b; ⬆ E-cadherin; ⬆ vimentin	[65]
2.	Clinical Trials NCT00433511	CTCs to predict late clinical recurrence in HER2- BC	LB – CTCs (peripheral blood)	547 Lymph Node Positive and High Risk Lymph Node Negative BC	CellSearch system for CTC detection	higher recurrence risk is associated with higher CTC burden.	[75]
3.	The LiqBreasTrack cohort study (Nov 2016-Feb 21)	Progression-free survival (PFS) between the first T-DM1 administration and progressive disease or last follow-up.	LB – ctDNA (plasma)	28 BC tissues (*n* = 14); 337 plasma (*n* = 20)	Targeted NGS and dPCR	⬆ MYC/FGFR1/ESR1 amplifications; ⬇ HER2 amplifications; ⬇ PIK3CA mutations	[79]
4.	Long non-coding RNAs	Diagnosis of early stage BC and patients with larger tumors (> 2 cm)	LB – lincRNA:miRNA (blood)	25 controls and 70 BC patients	q-PCR	⬆ LINC00511 and onco-miR-301a-3p; ⬇ miR-185-3p	[92]
5.	Circulating microRNAs	Predict the CTC status of patients with MBC	LB –Ct miRNAs (plasma)	269 samples (61 CTC-positive, 72 CTC-negative, 60 CTC-low MBC cases, and 76 controls)	TaqMan Human MicroRNA array	⬆ miR-141, miR-200a, miR-200b, miR-200c, miR-203, miR-210, miR-375, and miR-801 upregulated in CTC+ MBC; ⬇ miR-768-3p in MBC;	[99]
6.	SUCCESS A trial – MicroRNAs as prognostic markers in post-operative EBC patients	Associate whole blood miRNA profiles, presence of circulating tumor cells (CTCs) and clinical outcome in post-op EBC patients	LB – whole blood (pre-adjuvant therapy, post-adjuvant therapy, 2 years follow up)	48 post-operative patients; 17 female healthy donors as negative controls	miRNA quantitative PCR	⬆ miR-19a, miR-22 and miR-127 in EBCs; ⬆ miR-127 correlated with the presence of CTCs	[101]
7.	Precursor miRNAs of oncomiRs (miR526b and miR655)	Differentiate malignant tumors from benign lesions	LB – pri-miRNA (plasma)	90 malignant tumors; 20 benign lesions (control)	q-PCR	⬆ pri-miR526b and pri-miR655 in Stage I plasma and malignancy	[102, 103]
8.	4-circulatory miRNA signature in plasma of EBC	Early detection of (ER/PR+) IDC-BC from healthy controls	LB – miRNAs (plasma)	41 early invasive ductal carcinoma (IDC) Lebanese BC patients; 32 healthy controls	miRNA quantitative PCR	⬆ miR-145, miR-139-5p, miR-425-5p, and miR-130a upregulated in EBC plasma	[106]
9.	5-miRNA signature	Discern high- and low-risk of relapse (early recurrence and those with no recurrence) in post-operative BC patients	MiRNAs - Tumor (tissue sample)	71 primary BC (group A – disease-free); (group B - early relapse); (group C - late relapse)	Microarray and RT-qPCR validation	⬇ miR-149, miR-10a, miR-20b, miR-30a-3p and miR-342-5p in high-risk, early recurrence group	[108]
10.	Exosomal miRNAs	Diagnostic markers for BC	LB – miRs (plasma)	16 healthy plasma and 16 BC plasma	NGS, q-PCR	⬆ miR-1246 and miR-21	[117]
11.	Exosomal miRNAs	Diagnostic markers for BC subtypes	LB – miRs (serum)	Control (n-16); LA (n-16); TNBC (n-16)	RNA-seq; q-PCR	⬆ miR-142-5p, miR-320a and miR-4433b-5p	[118]
12.	Exosomal miRNAs	The diagnostic role of miR-106a-363 cluster in BC	LB - miRs (plasma, serum)	(400 plasma) - 200 BC patients and 200 healthy controls (HCs); (406 serum) - 204 BC patients and 202 HCs	q-PCR	⬆ plasma miRNAs (miR-106a-3p, miR-106a-5p, miR-20b-5p, miR-92a-2-5p); ⬆ serum miRNAs (miR-106a-5p, miR-19b-3p, miR-20b-5p, and miR-92a-3p)	[119]
13.	Circulating miRNAs	Diagnosis of early BC	LB - miRs (plasma)	65 BC patients and 34 HCs	real-time PCR (RQPCR)	⬆ miR-99a, miR-130a, miR-484 and miR-1260a	[121]
14.	Circulating miRNAs	Early detection markers for breast cancer	LB – miRs (plasma)	127 sporadic breast cancer cases and 80 healthy controls	TaqMan low-density arrays (TLDA) and qPCR)	⬆ miR-148b, miR-409-3p and miR-801	[122]
15.	Circulating miRNAs	Detect malignant from benign pre-treatment plasma of BC	LB – miRs (plasma)	Benign - 40; Non-metastatic BC – 54; Metastatic BC - 14	Microarray	⬆ hsa-miR-652-5p	[123]
16.	Circulating miRNAs	Early stage BC	LB – miRs (blood)	83 BC patients; 63 Healthy controls	real-time PCR (RQPCR)	⬆ miR-195	[124]
17.	Circulating miRNAs	Early stage BC	LB – miRs (serum)	210 BC patients; 102 Healthy controls	real-time PCR (RQPCR)	⬆ miR-195	[125]
18.	Circulating Biomarkers (miRNA+ protein)	ER+ BC to Bone metastasis	LB – exosomal miRs (blood)	23 ER+ BC patients; 22 healthy donors	Microarray; Western blot	⬆ exosomal miR-19a and Integrin-Binding Sialoprotein (IBSP)	[130]
19.	The NeoALTTO Study cohort	Pathological complete response (pCR) to single agent trastuzumab-based neoadjuvant therapy (NAC).	LB – miRs (plasma)	52 BC patients; 752 microRNAs	PCR assay	⬆ ct-miR-148a-3p, ⬆ ct-miR-374a-5p; ⬆ ct-miR-140-5p	[131]
20.	TNBC Exosomal miRs	Identify refractory and poor NAC responders	LB – miRs (Blood)	200 BC patients	droplet digital PCR (ddPCR)	⬇ miR-185, miR-4283, miR-5008, miR-3613; ⬆ miR-1302, miR-4715, miR-3144	[132]
21.	Circulatory miRNAs	Detect early stage BC	LB – miRs (serum)	1280 BC serum; 2836 non-cancer control; 63 non-breast benign samples	Microarray	⬆ miR-1246, miR-1307-3p, miR-4634, miR-6861-5p and miR-6875-5p)	[133]
22.	SUCCESS trial	Association of plasma miRs and BC invasiveness	LB – miRs (plasma)	111 BC patients (pre & post chemo); 46 Healthy controls	q-PCR	⬆ miR-16, miR-27a and miR-132 (pre-chemo); ⬇ miR-27a and miR-132 (post-chemo); ⬆ miR-107 (ER- tumors)	[134]
23.	Circulating biomarker (miRNAs + proteins)	Prognostic biomarkers of BC	LB – miRs; proteins (plasma)	30 BC patients; 10 HCs; 253 BC plasma samples	droplet digital PCR (ddPCR)	⬆ miR-923 and CA 15–3	[135]
24.	Circulating tumor cells (CTCs) and serum exosomal miRNAs	Diagnostic and predictive biomarkers of relapse post-NAC	LB – CTCs; miRs (serum)	53 post-NAC BC patients; 8 HCs; 6 metastatic patients from the NAC cohort	qPCR	⬆ miRNA-21, miRNA-222 and miRNA-155 correlate with CTCs in Metastatic patients	[136]

### MiRNA mediated targeted therapy against CTCs

Over the years, the enumeration of CTCs-miRNAs seems promising with an emerging trend of multiple clinical studies reporting miRNAs secreted by CTCs in liquid biopsies. The challenge is that the therapeutic value depends on the utilization of BC-specific miRNAs for accurate prediction and functional reversal of the breast cancer proliferation and metastases [137]. In a previous analysis where post-screening of 436 breast tumor tissue miRNAs was performed, miR-183 was the sole “CTC-specific” miRNA with high expression levels in patient blood samples having CTCs ≥5 /7.5 mL [51, 112]. CTCs display heterogeneous expression profiles of miR-10b, which is a regulator of viability in MBCs with effect in inducing miRNA-mediated targeted elimination of metastatic tumor cells [138, 139]. MiRNAs are crucial not only for the detection of CTC clusters, but are also key in efficiently targeting CTCs. Despite EMT being known as a pre-requisite for migration and metastases, evidence illustrates that BC metastases to lungs is more commonly enriched with non-EMT CTCs which successfully retain the typical epithelial morphology [140]. The formation of recurrent lung metastasis post-chemotherapy is mainly attributed to an acquired resistance and lower rate of proliferation in EMT cells to survive cyclophosphamide treatment. An overexpression of miR-200 abolished this chemoresistance in EMT prone cells indicating the potential utility of anti-miRNA therapy in combination with conventional chemotherapy regimen for treating aggressive BCs [140].

The presence of the primary unprocessed miRNA transcript pri-miR526b and pri-miR655 in the plasma of cancer patients effectively distinguished ER+ and HER2-fractions from ER- and HER2+ respectively, making them useful as adjuncts to current conventional endocrine therapy [101]. In TNBC and HER2+ disease, metastases to the brain is the most common early onset scenario. The advantages of focusing on secretory miRNAs is that the variations in expression levels is in accordance with BM progression, thus enabling sequential monitoring of the disease burden, response to therapy, and for accurate differentiation of brain lesions (BL) and metastatic deposits [31]. Mir-10, miR-21, miR-223, miR-711, miR-125, and miR-935 signatures in the cerebro-spinal fluid (CSF) have clearly discriminated the malignancies of central nervous system (CNS) from the metastatic deposits originating from breast and lung carcinoma [31, 141]. A miRNA cluster family (miR-93/20b/106a/106b) formed the core of the regulatory network that co-ordinated the suppression of multiple lncRNAs (MESTIT1, LOC100128164, and DNMBP-AS1) which are significantly associated with poor overall survival in breast cancer patients [142]. Combinatorial targeting of these key non-coding RNAs may provide new therapeutic strategies to prevent the progression of BC from an early-stage to advanced stage disease.

## Limitations and future implications

Metastasis is a rate–limiting progressive process with miRNAs acting as upstream master regulators of oncogenes and tumor suppressor genes. Despite the vast knowledge on the mutational profile of breast tumors and the subsequent events of invasion and metastases, there remains a paucity of data linking genetic mutations and miRNA expression profiles in primary and metastatic tumor cells. Yet to be addressed are scientific questions: (i) Does any of the major driver mutations occur in the genomic regions coding for miRNAs that are crucial in regulating metastases? (ii) How changes in miRNA expression levels impact the phenotype and cell fate of CTCs originating from EBCs and advanced BCs? (iii) Are systemically expressed miRNAs quantifiable representatives of tumor or are they host-related factors?

The lack of consensus between the different ct-miRNA signatures experimentally validated by various groups from the plasma samples of BC patients at various stages of the disease also raises concerns: (i) Why are there no uniform miRNA expression signatures reported in BC? (ii) Are the research findings reproducible across BC patients in different regions and among differing ethnic communities? (iii) Are there other external factors such as diet, immunological, and stress-related phenomena that tend to overpower the disease-specificity of these biomarkers in circulation? and finally, (iv) Do we need to follow a standard experimental protocol for retrieving a universally acceptable miRNA signature? Progress in screening techniques that warrants specificity and consistency is mandatory for the identification of novel cell-free miRs in circulation that is disease specific, reflects the tissue-of-origin and is measureable at significant levels in any sampling conditions.

Despite the secretion of miRNAs from a number of sources including PTs, MBCs, or CTCs in circulation, the expression levels might be reduced by a plethora of host and environmental factors. Thus the detection of miRNA signatures from within the CTCs warrant accuracy in enumeration and quantification of CTCs. A novel technology SILVER-seq (Small Input Liquid Volume Extracellular RNA Sequencing) can efficiently sequence both cell-specific and circulating fragmented exRNAs from a small volume (5 μL to 7 μL) of input serum liquid biopsy sample and even quantify donor-to-donor variations [143]. This offers the advantages of higher sensitivity, a less biased view of the small RNA transcriptome, as well as an increase in the number of putative miRNAs with prospective implications in clinical management of breast cancer. Designing novel techniques to enhance the accuracy in detection of even nano-levels of miRNAs is highly recommended.

## Data Availability

Not applicable.

## Abbreviations

LB: Liquid biopsy
EBC: Early breast cancer
MBC: Metastatic breast cancer
RNA: Ribonucleic acid
miR: MicroRNA
mRNA: Messenger RNA
DNA: Deoxy-ribonucleic acid
CTC: Circulating tumor cell
cfcNA: Cell-free circulating nucleic acid
DFS: Disease free survival
CEA: Carcinoembryonic antigen
CA: Cancer antigen
ELISA: Enzyme-linked Immunosorbent Assay
uPA: Urokinase plasminogen activator
PAI-1: Plasminogen activator inhibitor type-1
IF: Interstitial fluid
TME: Tumor microenvironment
MRD: Minimal residual disease
DTCs: Disseminated tumor cells
NOWAC: The Norwegian Women and Cancer (NOWAC) Post-Genome study
SNPs: Single nucleotide polymorphisms
EMT: Epithelial mesenchymal transition
PIPs: Progesterone-induced paracrine signals
CK14: Cytoskeletal keratin 14
EL: Early lesion
Ereg: Epiregulin
Tnc: Tenascin C
Jag1: Jagged1
MHC: Major histocompatibility
PFS: Progression-free survival
IBC: Inflammatory breast cancer
NAC: Neo-adjuvant chemotherapy
pCR: Pathological complete response
pri-miRNA: Primary microRNA
pre-miRNA: Precursor miRNA
t-RNA: Transfer RNA
rRNA: Ribosomal RNA
PBMC: Peripheral blood mononucleated cell
NKcells: Natural killer cells
CNA: Copy number aberrations
CTM: Circulating tumor emboli
AI: Artificial intelligence
BL: Brain lesions
BM: Brain metastases
CSF: Cerebro-spinal fluid
CNS: Central nervous system
NGS: Next-generation sequencing
